# Help-seeking behaviour and associated factors among women who experienced physical and sexual violence in Ethiopia: evidence from the 2016 Ethiopia Demographic and Health Survey

**DOI:** 10.1186/s12905-021-01574-0

**Published:** 2021-12-25

**Authors:** Simegnew Handebo, Ayenew Kassie, Adane Nigusie

**Affiliations:** 1 School of Public Health, St. Paul’s Hospital Millennium Medical College, Addis Ababa, Ethiopia; 2 Department of Health Education and Behavioural Sciences, Institute of Public Health, College of Medicine and Health Sciences, University of Gondar, Gondar, Ethiopia

**Keywords:** Help-seeking, Women, Violence, Ethiopia

## Abstract

**Background:**

Violence against women remains devastatingly pervasive and remained unchanged over the past decade. Violence against women is preventable and help-seeking of women subjected to violence is an entry point. So, this study assessed help-seeking behaviour and associated factors among women who experienced physical and sexual violence in Ethiopia.

**Method:**

Using the 2016 Ethiopia Demographic and Health Surveys (EDHS), this paper analyzes the determinants of help-seeking behavior of women subjected to violence in Ethiopia. EDHS used a two-stage stratified cluster sampling technique. From 642 communities, a total of 1540 (weighted) reproductive age women were included in the analysis. Simple descriptive, bivariable and multivariable logistic regression analysis were employed. Statistical significance was set at a *p*-value of less than 0.05.

**Results:**

Only 22.5% of the women who experienced violence sought help. Being aged 30 and above, working in sales, or an agricultural job, being in the richest wealth quintile, and experiencing severe violence were associated with increased help-seeking behaviour. Living in a rural area, having a husband who attended primary, secondary, and higher education, having a husband working in a professional job and agriculture were factors associated with lower odds of help-seeking behaviour.

**Conclusion:**

In Ethiopia below one-fourth of women who were subject to violence sought help. Socio-demographic factors and severity of the violence were associated with help-seeking. Preventing child marriages and reducing poverty as well as increased employment and education for women enhance help-seeking behaviour by the women. Interventions could include creating awareness, law enforcement, and support for victims.

## Background

Globally, more than 1.6 million people die as a result of violence each year [1]. Many more people are harmed and suffer from a variety of physical, sexual, reproductive, and mental health issues for every person who dies as a result of violence [2, 3]. Violence is one of the leading causes of death among people aged 15–44 years old around the world, accounting for about 14% of male deaths and 7% of female deaths [4]. The immediate and long standing effects of violence have been extensively investigated around the world, and they include psychological stress, family and community upheaval, economic loss, sickness, and mortality [5]. Increased rates of depression, anxiety, posttraumatic stress disorder, and suicide are all biological effects of violence [6].

According to studies, 15% and 71% of ever-partnered women have been physically or sexually assaulted by an intimate partner at some point in their lives [7]. In many countries, a substantial proportion of women who experienced physical violence also experienced sexual abuse [2]. According to studies in Mexico and the United States, 40–52% of women who are physically abused by an intimate partner are also sexually abused [1]. Various levels of intimate partner violence (IPV) prevalence have been recorded in Africa. The lifetime prevalence of IPV was 10.1% in Namibia, 50.9% in Ghana, and 61% in Tanzania [8–10]. IPV prevalence in Ethiopia varies from 32.5 to 78% [11–14].

Despite the fact that violence has always existed, the world does not have to accept it as an unavoidable aspect of life [15]. When violence is experienced, seeking help has a significant impact in reducing the repercussions of violence [16]. However, the presence of various barriers makes seeking assistance difficult [17]. Different studies identified factors that are associated with seeking help during the time of violence [18]. Factors such as unemployment, low educational status, economic dependence, and repeated previous violence have been associated with staying in an abusive relationship. The age, education level, and severity of the injury all influence help seeking behavior [19–21]. In addition, the length of marriage and the number of children has been linked with help seeking practice [20].

Though there are studies on the prevalence of violence, the issue of help-seeking behavior to terminate the violence and reduce its impact has generally been neglected. This study wants to examine help-seeking behavior of women who experienced physical and sexual violence. Some countries have investigated help-seeking behavior amongst women exposed to violence [20–23]. However, this has not been studied in Ethiopia. The aim of this study was to assess the magnitude and determinants of help-seeking behavior of reproductive age women who experienced physical and sexual violence in Ethiopia.

## Methods

### Study design, period and data sources

The data for this study was extracted from the 2016 EDHS dataset, which is publicly available from the Measure DHS website (http://www.measuredhs.com). The 2016 EDHS is the fourth and most recent in the Demographic and Health Survey in Ethiopia. The survey was community-based cross-sectional study conducted from January 18 to June 27, 2016 in nine regional states and two city administrations of Ethiopia [24]. Ethiopia is the second largest populous country in Africa with 102.4 million people and an annual population growth rate of 2.5%.

### Sampling and population

A two-stage stratified cluster sampling technique was employed. In the first stage, Enumeration Areas (EAs) were selected. In the second stage, 28 households per EAs were selected. A total of 645 EAs (202 in urban areas and 443 in rural areas) were selected with probability proportional to EAs size. All women age 15–49 years who were either permanent residents of the selected households or visitors who stayed in the household the night before the survey were eligible to be interviewed. A total of 15,683 reproductive women were interviewed and 1540 (weighted) women who experienced physical and sexual violence were included in the final analysis [24].

### Outcome variable

Help-seeking behavior of women who experienced physical or sexual violence was the outcome variable. Women who experienced violence were asked whether they have ever sought help to stop violence or not; and responses were coded as 1 if “yes” 0 if “no”. The study adopted the response to this question to assess factors associated with help-seeking behavior among of reproductive age women.

### Independent variables

The independent variables were selected based on the previous literature and availability of the variable in the 2016 EDHS dataset. These were age of woman, educational status, occupational status, place of residence, religion, husband occupation, husband educational status, administrative region, wealth index, media exposure, severity of injury, number of live births, and duration of the marriage.

### Operational definition

- Administrative region was recoded as ‘Cities’ (which include Addis Ababa, Harari and Dire Dawa), ‘Main region’ (Tigray, Amhara, Oromia, and SNNPR), and ‘Pastoral’ (which include Afar, Benshangul-Gumuz, Gambela and Somali).
- Media use was recoded as reading newspaper or listening to radio, or watching television.
- Respondent occupation was recoded as not working, professional (sales, professional, and clerical), agriculture, and others (skilled, unskilled, service and other)
- Husband occupation was recoded as not working, professional (professional, sales, service, clerical, skilled and service), agricultural, unskilled, and others (others and don’t know).

### Statistical analysis

The data was analyzed using STATA version 14. Sample weights were applied to compensate for the unequal probability of selection between the strata and non-responses. A detailed explanation of the weighting procedure can be found in the EDHS methodology report [25]. Descriptive analysis was performed to show the distribution of sociodemographic characteristics of participants and prevalence of help-seeking behavior. Bivariable and multivariable logistic regression was employed to identify the determinants of help-seeking behavior. A *p*-value less than 0.25 in bivariable logistic regression were selected for multivariable logistic regression. Both crude and Adjusted odds ratio (AOR) and 95% Confidence Interval (CI) were used to assess the strength of associations between the outcome and the independent variables. The threshold for statistical significance was set at *p* < 0.05. *p*-value less than 0.25 in bivariable logistic regression were selected for multivariable logistic regression.

## Results

### Characteristics of the respondents

From 2016 EDHS dataset, a total of 1540 reproductive age women who experienced physical or sexual violence were included for this study. The mean age of women was 30.58 (SD ± 8.79) years old. Nearly half (48%) of women were Orthodox Christian by religion. Majority (80.8%) of the women were rural residents and 58.5% had no formal education (Table 1).

**Table 1:** Socio-demographic characteristics of women experienced physical and sexual violence in Ethiopia (n = 1540)

Variable	Category	Frequency	Percent
Age	15–19	143	9.30
	20–24	262	17.03
	25–29	312	20.28
	30–49	823	53.39
Residence	Urban	295	19.18
	Rural	1245	80.82
Religion	Orthodox	740	48.02
	Protestant	333	21.63
	Muslim	425	27.60
	Other	42	2.75
Region	Major	1427	92.67
	Pastoral	13	0.85
	Cities	100	6.48
Educational status	No education	900	58.47
	Primary	460	29.86
	Secondary	120	7.79
	Diploma and above	60	3.88
Occupation	Not working	686	44.51
	Professional	294	19.06
	Agriculture	371	24.09
	Other	190	12.34
Husband education	No education	596	50.50
	Primary	433	36.73
	Secondary	85	7.22
	Higher	53	4.48
	don't know	13	1.08
Husband occupation	Not working	88	7.45
	Professional	231	19.56
	Agriculture	782	66.27
	Unskilled	35	2.96
	Other	44	3.76
Number of live children	0	212	13.76
	1–2	490	31.84
	3–4	384	24.94
	≥ 5	454	29.46
Wealth index	Poorest	309	20.09
	Poorer	266	17.25
	Middle	362	23.49
	Richer	302	19.61
	richest	301	19.56
Media exposure	No	930	60.37
	Yes	610	39.63

### Help-seeking behaviour

**Fig. 1:**
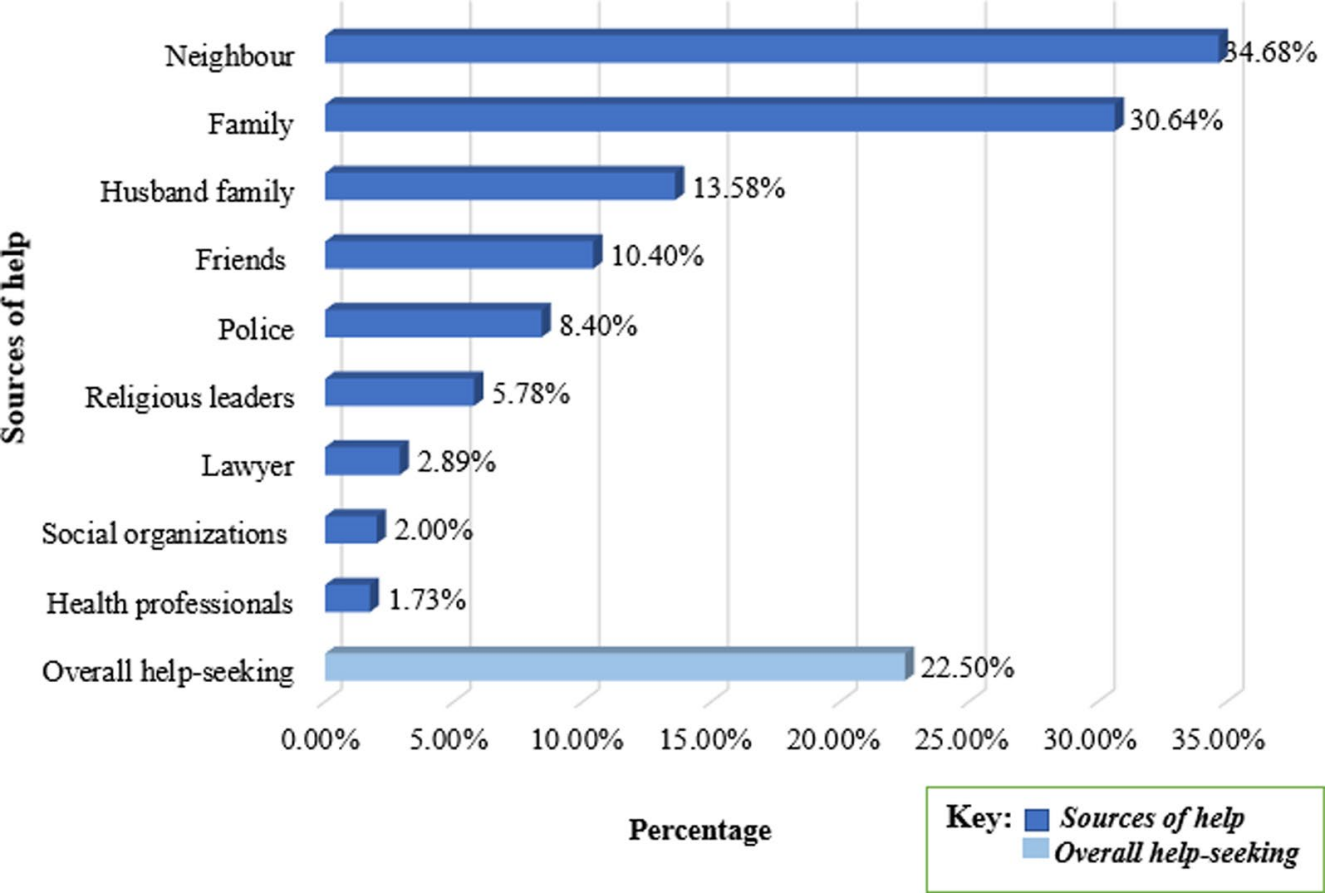
Over all help-seeking behavior and sources of help among women experienced physical and sexual violence in Ethiopia

Of women experienced violence, 22.45% (95% CI 20.43–24.61%) sought help. From those women about 119 (34.7%) sought from a neighbour, 106 (30.6%) sought help from their family, and 47(13.6%) sought from their husband’s family member. Of 168 (10.9%) currently pregnant women experienced violence, only 27 (16.1%) of them sought help. On the other hand, for women who did not seek help, 174 (14.54%) told someone about the violence after some time (Fig. 1).

### Determinants of violence

Bivariable logistic regression was performed to see the association between help-seeking behaviour of women with predictor variables. In the multivariable logistic regression analysis; age, residence, occupation, husband education, husband occupation, wealth index, and severity of the injury were significantly associated with help-seeking behaviour. Table 2 shows crude and adjusted odds ratios that were calculated to determine the strength of association between the co-variates and help-seeking behaviour.

**Table 2:** Factors associated with help-seeking behavior among women experienced physical and sexual violence in Ethiopia (n = 1540)

Variable	Category	Help seeking		COR (95%)	AOR (95%)
		Yes (%)	No (%)		
Age	15–19	36 (24.78)	108 (75.22)	1	1
	20–24	33 (12.50)	229 (87.50)	0.43 (0.26,0.73)	2.32 (0.42, 12.64)
	25–29	52 (16.70)	260 (83.30)	0.61 (0.38, 0.98)	2.50 (0.42, 14.74)
	30–49	225 (27.41)	597 (72.59)	1.15 (0.76, 1.72)	8.69 (1.43, 52.68)*
Residence	Urban	240 (19.30)	1005 (80.7)	1	1
	Rural	106 (35.76)	190 (64.24)	0.43 (0.33, 0.57)	0.30 (0.15, 0.57)**
Duration of marriage	Less than 2 years	70 (19.45)	290 (80.5)	1	1
	2–5 years	185 (23.66)	595 (76.34)	1.28 (0.94, 1.75)	1.21 (0.57, 2.57)
	Greater than 5 years	91 (22.45)	309 (77.21)	1.22 (0.86, 1.73)	0.59 (0.25, 1.38)
Religion	Orthodox	182 (24.54)	558 (75.46)	1	1
	Protestant	77 (23.21)	256 (76.79)	0.93 (0.69, 1.26)	1.25 (0.80, 1.95)
	Muslim	83 (19.58)	342 (80.42)	0.75 (0.56, 1.00)	1.10 (0.72, 1.68)
	Others	4 (8.95)	39 (91.05)	0.30 (0.10, 0.88)	0.47 (0.14, 1.59)
Region	Major	306 (21.44)	1121 (78.56)	1	1
	Pastoral	1 (10.06)	12 (89.44)	0.41 (0.07, 2.49)	0.20 (0.01, 2.76)
	Cities	39 (38.60)	61 (61.40)	2.30 (1.51, 3.51)	0.69 (0.30, 1.62)
Educational status	No education	198 (21.99)	702 (78.01)	1	1
	Primary	89 (19.34)	71 (80.66)	0.85 (0.64, 1.12)	0.87 (0.55, 1.38)
	Secondary	41 (34.20)	79 (65.80)	1.84 (1.22, 2.77)	1.96 (0.87, 4.41)
	Above secondary	18 (29.78)	42 (70.22)	1.50 (0.85, 2.68)	0.94 (0.31, 2.89)
Occupation	Not working	131 (19.09)	555 (80.91)	1	1
	Professional	79 (30.83)	178 (69.17)	1.79 (1.31, 2.45)	2.15 (1.32, 3.49)**
	Agriculture	88 (21.74)	319 (78.26)	1.18 (0.86, 1.61)	1.68 (1.09, 2.56)*
	Other	47 (24.77)	143 (75.23)	1.40 (0.95, 2.04)	0.99 (0.54, 1.81)
Husband education	No education	139 (23.26)	457 (76.74)	1	1
	Primary	73 (16.83)	360 (83.17)	0.67 (0.49, 0.92)	0.53 (0.35, 0.79)**
	Secondary	13 (15.09)	72 (84.91)	0.59 (0.31, 1.09)	0.39 (0.17, 0.88)*
	Higher	11 (21.66)	41 (78.34)	0.91 (0.46, 1.80)	0.37 (0.15, 0.95)*
	Don't know	3 (19.69)	10 (80.31)	0.81 (0.20, 3.27)	0.38 (0.07, 1.97)
Husband occupation	Not working	31 (35.72)	57 (64.28)	1	1
	Professional	57 (24.86)	173 (75.14)	0.60 (0.35, 1.01)	0.48 (0.24, 0.96)*
	Agriculture	129 (16.51)	653 (83.49)	0.36 (0.22, 0.57)	0.36 (0.20, 0.63)**
	Unskilled	11 (32.82)	23 (67.18)	0.88 (0.38, 2.02)	0.89 (0.32, 2.48)
	Other	9 (20.30)	35 (79.70)	0.46 (0.20, 1.07)	0.38 (0.13, 1.05)
Number of live children	No child	50 (23.61)	162 (76.39)	1	1
	1–2	109 (22.31)	381 (77.69)	0.93 (0.63, 1.36)	1.06 (0.36, 3.13)
	3–4	81 (21.07)	303 (78.93)	0.86 (0.58, 1.29)	0.84 (0.26, 2.70)
	≥ 5	105 (23.25)	348 (76.75)	0.98 (0.67, 1.44)	0.93 (0.28, 3.02)
Wealth index	Poorest	73 (23.62)	236 (76.38)	1	1
	Poorer	67 (25.36)	198 (74.64)	1.10 (0.75, 1.61)	1.25 (0.75, 2.11)
	Middle	64 (17.83)	297 (82.17)	0.70 (0.48, 1.02)	0.87 (0.52, 1.46)
	Richer	60 (19.81)	242 (80.19)	0.80 (0.54, 1.17)	1.23 (0.72, 2.09)
	Richest	81 (26.90)	220 (73.10)	1.19 (0.83, 1.71)	1.76 (1.02, 3.04)*
Media exposure	No	192 (20.69)	738 (79.31)	1	1
	Yes	153 (25.14)	457 (74.86)	1.29 (1.01, 1.64)	0.73 (0.48, 1.09)
Severity of violence	Mild	191 (16.44)	973 (83.56)	1	1
	Serious	114 (44.38)	142 (55.62)	4.10 (3.03, 5.42)	5.19 (3.56, 7.58)**

^*^Significant at *p* value < 0.05

^**^Significant at *p* value < 0.001

After adjusting for other variables, women whose age was 30 years and above were 8.69 times more likely to seek help than women aged 15–19 years old (AOR = 8.69, 95% CI 1.43, 52.68). The odds of help-seeking behavior of rural area resident women decreased by 70% compared to urban residents (AOR = 0.30, 95% CI 0.15, 0.57). The likelihood of help-seeking behaviour was higher among women working sales (AOR = 2.15, 95% CI 1.32, 3.49) and agriculture (AOR = 1.68, 95% CI 1.09, 2.56) compared to women with had no work. The odds of help-seeking behaviour decreased among women whose husband attended primary (AOR = 0.53, 95% CI 0.35, 0.79), secondary (AOR = 0.39, 95% CI 0.17, 0.88) and higher (AOR = 0.37, 95% CI 0.15, 0.95) education compared to women who had husbands with no education. Women whose husbands were working a professional job (AOR = 0.48, 95% CI 0.24, 0.96) and agriculture (AOR = 0.36, 95% CI 0.20, 0.63) were 52% and 64% less likely to seek help than unemployed husbands, respectively. Women in richest wealth quintiles (AOR = 1.76, 95% CI 1.02, 3.04) were nearly twice as likely to seek help as women in poorer wealth quintiles. The odds of help-seeking behaviour of women who experienced serious violence were 5.19 (AOR = 5.19; 95% CI 3.56, 7.58) times higher than women experienced mild violence (Table 2).

## Discussion

This article highlighted the proportion of help-seeking behavior among women subjected to violence in Ethiopia. In addition, it has contributed to the literature in the area of violence related help-seeking practice. This study found that less than a quarter (22.5%) of women who experienced violence sought help, which is in-line with a study completed in India [23], demonstrating that most female victims of violence did not seek help. This result is higher than findings in Mali (17.6%) [23], Dhaka Slums (19%) [21], and Afghanistan (20%) [26]. But lower than other developing nations [23], and a study done Nigeria (39.7%) [22], and Uganda (63.5%) [27]. The discrepancy may be due to the difference in religious and cultural views to violence [28]. On the other hand, violence within the marriage is considered as a family matter [20].

We found that older women (30 years and above) were more likely to seek help compared to their younger counterparts. This finding is in line with a study done in developing countries [23] and Canada [29]. A study in Afghanistan revealed women aged 25–34 years old more likely seek help than the youngest group [26]. However, Roberto et al. [30] reported that older women were less likely to report help seeking than their younger counterparts. This may be because older women know about sources of help and develop confidence to report the violence that occurs [29]. However, in some cases, as the age of the women increases and they have been in a relationship longer, they became more autonomous, and learn how to deal with the violence; this may contribute to a decrease in help-seeking behavior [31]. Studies completed in India, Nigeria, and Uganda did not show any significant association between age and help-seeking behavior [20, 22, 27].

In urban areas, various institutions are available to support women who seek help [32]. In support of this, the present study found that the help-seeking behavior of women living in rural areas was lower than those living in urban areas. In the rural areas, cultural and socioeconomic factors as well as accessibility of sources of help, may make help-seeking behavior difficult [28, 33]. Besides, rural women may consider violence as a private domain which is highly confidential [34]. This finding is different from studies done in Dhaka Slums and Bangladesh, in which rural dwellers had higher help seeking behavior than the urban women [21, 32]. This may be due to lengthy and costly legal processes which discourage urban resident women from seeking help [20].

The other factor that was found to be associated with help-seeking behavior was occupational status. Women employed in sales and agriculture jobs had higher help-seeking behavior. This may be due to employment which enable women to generate their own income and greater freedom to seek help [35]. Therefore, targeting women’s employment is an important intervention in the efforts to empower them and enhance help-seeking behavior to prevent and stop violence. On the other hand, having a husband with professional and agriculture jobs was associated with lower odds of seeking help. The odds of help-seeking behavior was lower among women whose husband had primary, secondary, and higher education compared to husbands with no education. The more educated the husband and the more professional the job, the less likely the woman will seek help. This may be because communication or cognitive skills gained from being more educated may contribute to better conflict resolution within marriage [37]. In addition, most often women are younger, not working, and less educated than their husbands, and the husband tends to dominate and discipline the wife [38]. Conversely, previous studies have revealed that women who had educated and employed husbands were less likely to experience violence [32, 36]. But the relationship between husband education and employment status and help-seeking for violence need further investigation.

Wealth status was found to be significantly associated with help-seeking behavior. Compared with the poorer wealth quintile, being in the richest quintile was associated with higher odds of seeking help for violence. Inversely, a study done in Canada reported that women with lower incomes were more likely to seek help than women with higher incomes [29]. Another study from India reported that women in the middle three wealth quintiles sought help than the poorest [20]. This implies that women with higher incomes are more likely to access resources to seek help [39]*.*

Women who experienced sever violence have higher odds of help-seeking behavior than those who experienced less severe (mild) violence. Similarly, evidences have found a positive association between severe violence and help-seeking behavior [20, 21, 29]. This might be because experience of severe violence is more easily identifiable while seeking help. On the other hand, it is somewhat difficult to disclose mild violence since they are not visible and might elicit different responses from the source of help [35, 40]. Unlike other studies [20, 29, 34]. We found that education status of women, duration of marriage, number of children, religion, and media exposure were not associated with help-seeking behavior.

This study has some strengths. First, it used nationally representative data which allowed generalization of findings. Sample weights were applied in all analysis. Despite its strength this study has some limitations. First, the data on violence and help seeking behavior was self-reported which may have recall and social desirability bias, leading to an under-reporting of the rates. The other limitation was that EDHS did not provide information about the context surrounding the women, women’s knowledge and attitude towards violence, help seeking strategies, cultural values and norms, and accessibility of facilities for seeking help for a violence. Furthermore, due to the nature of the cross-sectional study, causal relationship cannot be drawn. Therefore, future studies need to consider such socio-cultural and environmental factors and the way in which these factors shape women’s help-seeking behavior.

## Conclusion

In conclusion, this study examined help-seeking behavior of women who experienced physical and sexual violence in Ethiopia. Despite high prevalence of violence, a substantial proportion of women suffered from violence had very low help-seeking behavior. Socio-demographic factors like age, occupation, husband’s education and occupation status, wealth status and severity of the violence were associated with help seeking behavior. Prevention of child marriages, creating job opportunities, poverty reduction, and ensuring educational attainment of women are recommended interventions to enhance help-seeking behavior of women subjected to violence in Ethiopia. Legal and support institutions like police and healthcare services need to be readily accessible to reduce the impacts of violence. In addition, community-based education programs on women rights and legal supports are also mechanisms to enhance help-seeking behavior and help curb violence.

## Data Availability

The raw data used in this study can be accessed from the DHS website: http://www.dhsmeasures.

## Abbreviations

AOR: Adjusted odds ratio
CI: Confidence interval
CSA: Central Statistical Agency
EAs: Enumeration areas
EDHS: Ethiopia Demographic and Heath Surveys
HIV: Standard deviation
SNNPR: Southern Nations, Nationalities, and People’s Region
WHO: World Health Organization
